# Systematic techniques for assisting recruitment to trials (START): study protocol for embedded, randomized controlled trials

**DOI:** 10.1186/1745-6215-15-407

**Published:** 2014-10-25

**Authors:** Jo Rick, Jonathan Graffy, Peter Knapp, Nicola Small, David J Collier, Sandra Eldridge, Anne Kennedy, Chris Salisbury, Shaun Treweek, David Torgerson, Paul Wallace, Vichithranie Madurasinghe, Adwoa Hughes-Morley, Peter Bower

**Affiliations:** National Institute of Health Research (NIHR) School for Primary Care Research, Manchester Academic Health Science Centre, Centre for Primary Care, University of Manchester, Oxford Road, Manchester, M13 9PL UK; Department of Public Health and Primary Care, The Primary Care Unit, University of Cambridge, Cambridge, CB2 OSR UK; Department of Health Sciences, University of York, University Road, Heslington, York, YO10 5DD UK; William Harvey Research Institute, Barts and the London Queen Mary University of London, Charterhouse Square, London, EC1M 6BQ UK; Blizard Institute, Barts and The London School of Medicine and Dentistry, 4 Newark Street, London, E1 2AT UK; NIHR Collaboration for Leadership in Applied Health Research and Care (CLAHRC) Wessex, Health Sciences, University of Southampton, Highfield, Southampton, SO17 1BJ UK; Centre for Academic Primary Care, School of Social and Community Medicine, University of Bristol, 39 Whitely Road, Bristol, BS8 2PS UK; Health Services Research Unit, University of Aberdeen, Fosterhill, Aberdeen, AB25 2ZD UK; York Trials Unit, Department of Health Sciences, University of York, University Road, Heslington, York, YO10 5DD UK; NIHR Primary Care Research Network, Research Department of Primary Care and Population Health, University College London Medical School, Rowland Hill Street, London, NW3 2PF UK; Medical Research Council North West Hub for Trials Methodology Research, Manchester Academic Health Science Centre, Centre for Primary Care, University of Manchester, Oxford Road, Manchester, M13 9PL UK

**Keywords:** Randomized controlled trial, Recruitment, Primary care, Community, Trial participation, Intervention, Multimedia, Decision support systems, Protocols, Participant information

## Abstract

**Background:**

Randomized controlled trials play a central role in evidence-based practice, but recruitment of participants, and retention of them once in the trial, is challenging. Moreover, there is a dearth of evidence that research teams can use to inform the development of their recruitment and retention strategies. As with other healthcare initiatives, the fairest test of the effectiveness of a recruitment strategy is a trial comparing alternatives, which for recruitment would mean embedding a recruitment trial within an ongoing host trial. Systematic reviews indicate that such studies are rare. Embedded trials are largely delivered in an *ad hoc* way, with interventions almost always developed in isolation and tested in the context of a single host trial, limiting their ability to contribute to a body of evidence with regard to a single recruitment intervention and to researchers working in different contexts.

**Methods/Design:**

The Systematic Techniques for Assisting Recruitment to Trials (START) program is funded by the United Kingdom Medical Research Council (MRC) Methodology Research Programme to support the routine adoption of embedded trials to test standardized recruitment interventions across ongoing host trials. To achieve this aim, the program involves three interrelated work packages: (1) methodology - to develop guidelines for the design, analysis and reporting of embedded recruitment studies; (2) interventions - to develop effective and useful recruitment interventions; and (3) implementation - to recruit host trials and test interventions through embedded studies.

**Discussion:**

Successful completion of the START program will provide a model for a platform for the wider trials community to use to evaluate recruitment interventions or, potentially, other types of intervention linked to trial conduct. It will also increase the evidence base for two types of recruitment intervention.

**Trial registration:**

The START protocol covers the methodology for embedded trials. Each embedded trial is registered separately or as a substudy of the host trial.

## Background

Fundamental to health research is the testing of interventions through trials. Although many thousands of trials have been conducted, it is well known that achieving high levels of professional and patient participation in randomized controlled trials is often problematic. Many trials fail to recruit sufficient numbers of patients, or at least fail to do so in a timely fashion [1–4]. Other trials may reach recruitment targets by approaching large numbers of potential participants, but only recruit a small proportion of those actually eligible, with implications for both resources and bias. Recruitment problems can reduce the total recruited sample (limiting statistical power) and the proportion of eligible participants who participate (limiting external validity). Poor recruitment means a trial runs the risk of being underpowered and clinically relevant results may be reported as statistically non-significant, increasing the chance that an effective intervention will either be abandoned before its true value is established, or at the very least, delayed as further trials or meta-analyses are conducted. Recruitment is seen as the methodological research priority for clinical trials units in the United Kingdom [5].

Qualitative researchers have explored the recruitment process in depth [6–8], highlighting factors that could potentially act as levers for improving practice, including communications between lead researchers and other staff, and time and resource for governance. Key insights can be gained from such work, and can serve as the basis for the development of recruitment interventions [9, 10]. However, these designs are not suited for the evaluation of the effect of interventions on recruitment. For that, the most robust test of the effectiveness of a recruitment method is a trial comparing one method with an alternative, embedded in a real host trial. Embedding means that patients being recruited to an ongoing trial are additionally randomized to one of the two or more alternative recruitment strategies being evaluated. Such studies allow an unbiased assessment of the effectiveness of the recruitment intervention on a variety of recruitment outcomes, such as total numbers recruited, the proportion taking part, and the cost and efficiency of the recruitment process. Moreover, because the evaluation is embedded within an ongoing trial, the method evaluates the strategies in the context of a real decision to take part or not by potential participants.

The acceptance of the trial as the bedrock of outcomes research has led to a vast number of trials conducted (over 790,000 records on the Cochrane central register of controlled trials (CENTRAL) as of June 2014). Given the size of this potential platform for recruitment studies, and the consensus among the research community concerning the challenge of recruitment, it is surprising that embedded trials of recruitment interventions remain so rare. The Cochrane review on recruitment interventions identified only 45 embedded studies in real and hypothetical trials [11, 12] and concluded that ‘it would be better if more researchers included an evaluation of recruitment strategies in real trials’. The failure to grasp this opportunity means that recruitment for science is not underpinned by a science of recruitment.

Although a general increase in the number of embedded trials would be welcome, a more ambitious approach might extend the evidence base more systematically and rapidly. This would involve embedding the routine use of embedded recruitment trials in the research funding process, so that embedded trials are an accepted part of the delivery of all trials, adopted by a significant proportion of principal investigators as part of a wider endeavor across the trials community. This would be akin to the way in which patient and public involvement has become routinely embedded in health research [13]. Achieving this would not only rapidly develop the evidence base, it would also have the advantage of enabling similar recruitment interventions to be tested across multiple trials, allowing a clearer assessment of their general utility, and their sensitivity to contextual factors such as clinical populations, and interventions under test, setting, or time.

Although attractive in principle, the low frequency of use of embedded studies suggests that barriers exist. Qualitative work with key stakeholders (principal investigators, research managers, research ethics committee chairs, and funding representatives) has highlighted several challenges [14]. Although respondents recognized the case for embedded recruitment studies, their enthusiasm was tempered by a number of issues in implementing such studies in routine practice. Perceived challenges for host studies included increased management burden in addition to the testing requirements of the main trial, potential incompatibility between the host and embedded study, and the impact of the embedded study on trial design and relationships with collaborators. For embedded recruitment studies, there were concerns that host investigators might have strong preferences about one of the recruitment interventions under test, limiting the embedded study investigators’ control over the delivery of the embedded study. Overall, research on recruitment was welcomed in principle, but raised issues concerning control.

The START study (funded by the Medical Research Council (MRC) Methodology Research Programme) is designed to develop the conceptual, methodological, and logistical framework to make embedded recruitment trials a routine part of the delivery of trials, and to test this approach by developing a small number of recruitment interventions and testing them across multiple host trials. Our long-term aim is to support more efficient trials by improving the evidence base concerning recruitment to trials, and thus enhance recruitment rates, make better use of health research resources and, ultimately, support improvement in patient health.

### Aims

The aim of the START program is to develop a methodological framework and process of implementation for developing and testing embedded recruitment interventions within ongoing host trials. To achieve this, the program involves three work packages: (1) methodology - developing guidelines for the design, analysis and reporting of embedded studies, developing resources for those planning embedded studies, including, templates for embedded study protocols, embedded study registration, data sharing, and publication agreements; (2) interventions - developing recruitment interventions for embedding; and (3) implementation - recruitment of principal investigators of potential host trials, embedding of our recruitment interventions with host trials, and the testing of their effects on recruitment.

## Methods/Design

### Design overview

The initial focus of START is on the recruitment of patients in primary care and community settings, and in publically funded trials. This partly reflects the composition and expertise of the research team, and has influenced the choice of the initial interventions to be tested within START, as these are suited to the ‘remote’ strategies (such as postal surveys) often used to recruit patients into primary care and community trials, compared to the predominance of face-to-face strategies that are used in secondary care settings. However, the broad START approach will have wider relevance in a range of contexts and trial types.

### Work package 1: methodology

We will develop a framework for the evaluation of embedded recruitment interventions. This will involve a review of the design issues and statistical methods specific to studies of embedded recruitment interventions (for example, where the unit of analysis in host and embedded studies differ). We will extend the analytic framework to consider issues such as the analysis of the effects of recruitment interventions across different studies, sites, and practices. We will also develop guidelines for the measurement of the various outcomes of recruitment interventions such as: numbers of patients recruited, rate of recruitment, rate of retention, participant satisfaction, knowledge of the recruitment process, measures of informed consent and anxiety, sensitivity and/or specificity of recruitment methods in terms of proportions of eligible and/or ineligible patients identified, and the cost of recruitment interventions and the recruitment process.

We will develop a framework for the reporting of embedded recruitment studies, exploring the need for appropriate extensions to the Consolidated Standards of Reporting Trials (CONSORT) guidelines. The initial criteria will be drawn up by going through published reports of embedded trials assessing the recruitment interventions. Relevant studies will be identified by hand-searching the reference lists of earlier reviews [12, 15–18] on this topic, and contacting co-applicants for information on relevant studies known to them. The methodological review will be supported by structured searches of the literature, complemented by analysis of the methods used in published trials identified by the earlier reviews [12, 15–18].

### Work package 2: developing interventions

We chose to test the effects of two recruitment interventions for work package 2. Both interventions related to the provision of initial information for potential patients, as opposed to other key aspects of the recruitment process (such as recruitment of sites or professionals, or different methods of participant identification [19]). Our choice was based in part on a qualitative study of research staff perspectives, which found that ‘providing clear and concise information to potential participants’ was rated as ‘very important’ by 72% of respondents [20]. There is evidence that existing information in trials does not support high quality decision-making [21], and so the testing of enhancements is of high relevance. We also chose interventions that targeted the same aspect of the trial process (initial information), but which differed in content, cost, and complexity, to explore the yield of different methods which might differ in the ease with which they might be more widely implemented in trials.

However, the choice also reflected pragmatic issues. We had existing expertise in these interventions, and could draw on existing resources. The information provided to patients is readily modifiable, and such changes at the patient level can be allocated at random in embedded recruitment studies with comparative ease. In contrast, other embedded recruitment interventions, such as allocating patients to an initial approach from different professionals [22], or identifying patients using different methods, pose more significant logistical barriers, which may not be optimal for early phase feasibility work. Details of the intervention development process for the two interventions are described below.

#### Intervention 1: optimized participant information sheets

Research ethics committees rightly want to ensure that participants receive appropriate information and are able to provide fully informed consent. However, a long and complex participant information sheet (PIS) may impact negatively on recruitment, particularly if it is also visually unappealing or raises inappropriate levels of anxiety [21]. This is especially critical when patients are initially approached by a letter from their health professional, which occurs in a significant proportion of trials in primary care and community settings, especially in patients with long-term conditions. A systematic review has identified evidence that involving consumers in the development of patient information results in higher relevance and readability, without increasing anxiety [23].

**Figure 1:**
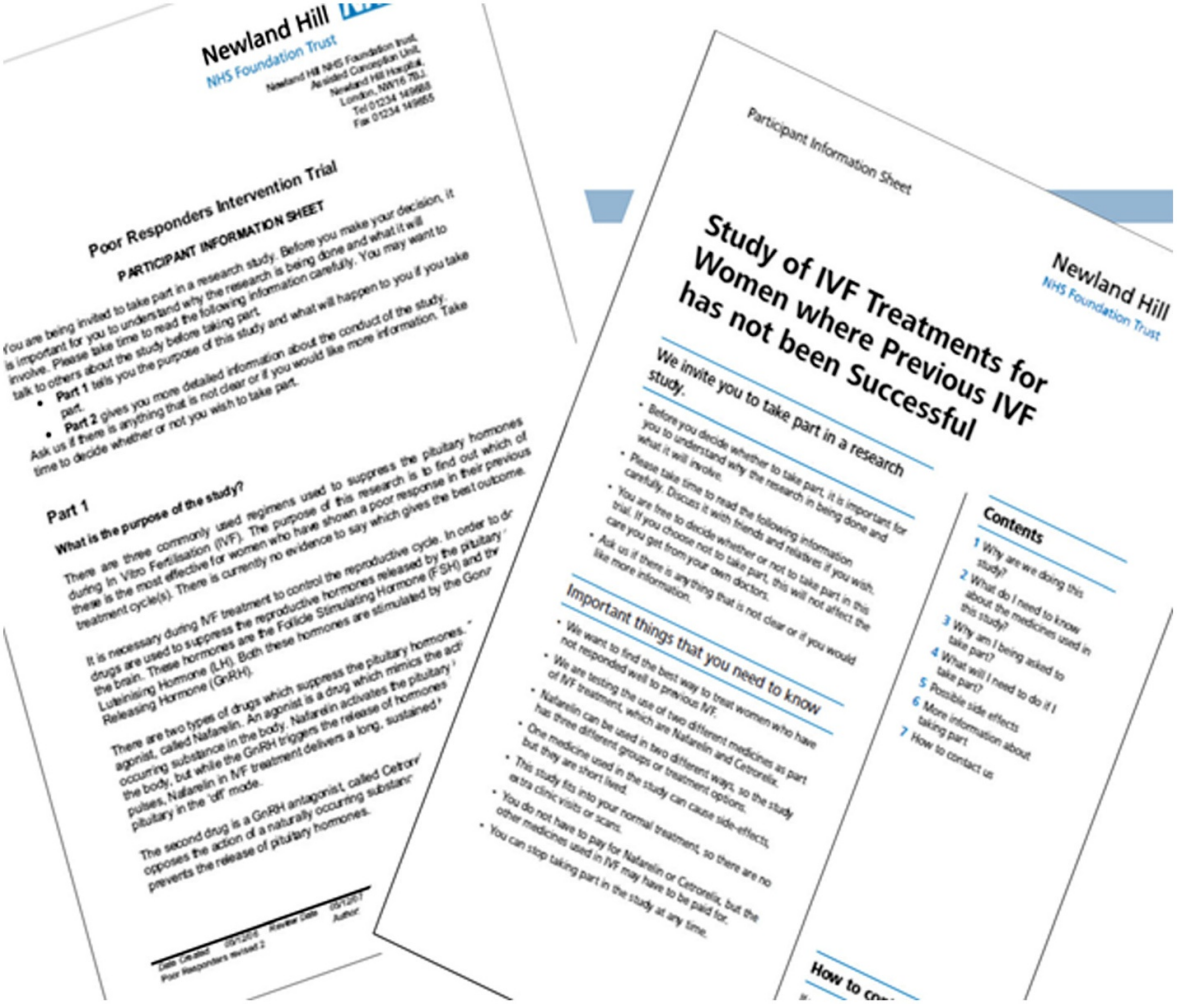
**Exemplar original (left) and optimized (right) participant information sheets** [25] **.**

To test the impact of involving consumers in the development of patient information, a revised version of the PIS will be prepared for each host trial. The process involves optimization of PIS readability through expertise in writing for patients and improved presentation via graphic design [24]. The revisions are informed by user testing [25–27], where the ability of patients to locate and understand key pieces of information is evaluated objectively to provide an assessment of the ability of the PIS to provide information in a way that can be understood. Initially the original PIS is tested, and then versions of the optimized PIS are tested (followed by further revision), until the resultant PIS is better able to inform potential trial participants. The optimized PIS would cover the same topics as the original version but the optimized version would likely differ in appearance, structure, and wording. See Figure 1 for an exemplar of an original and optimized PIS [25].

Members of the START research team have experience in this process, and user testing to develop the optimized PIS will be conducted through a commercial company (Luto Research Ltd, a University of Leeds (United Kingdom) spin-out company that provides information writing and testing services to the pharmaceutical industry). It is anticipated that, if successful, this intervention could be funded through additional grant funding for PIS optimization. Patients in each host trial will then be randomized to either the original or optimized PIS.

#### Intervention 2: multimedia concerning participation in research

At present most information about trial participation is presented in written form, but this is not necessarily the best way to communicate complex messages to all segments of the targeted population, particularly as so much communication now happens via the internet, often using video. Multimedia interventions may offer a useful strategy to improve communication about participation and may therefore facilitate greater accrual and retention rates. The diverse methods of information delivery possible via multimedia platforms provide alternative channels for health communication, in particular the internet provides an opportunity for self-directed and tailored learning [28–32]. However, the impact of multimedia information on patient-identified barriers and motivators to trial participation has not been rigorously explored.

We therefore set out to design and test multimedia approaches for delivering information to potential research participants alongside the standard, written PIS [33]. The design process was built on previous qualitative research [34, 35], and a review of research on patient decision-making conducted by the investigators (Hudson J, Rick J, Hughes-Morley A, Bower P: *What psychosocial factors are identified by patients as being important determinants of their decision to participate in clinical trials and can these be targeted by multimedia intervention? A meta-review*: unpublished data), and involved multiple iterative discussions within the research team. These deliberations covered the aims and content of the multimedia interventions, design issues to maximize relevance and impact, minimizing the burden on potential participants, how it would be evaluated, and ways to encourage potential participants to view the resources.

During the design process, the research team drew on several resources, to test the content and delivery platform for the intervention. Firstly, a workshop was conducted at the Primary Care Research Network (PCRN) national conference (November 2012), led by a clinician with considerable PCRN expertise and attended by PCRN research staff. Secondly, input and feedback was obtained from two Patient and Public Involvement representatives from the University of Manchester, Primary Care Research in Manchester Engagement Resource (PRIMER [36]). Thirdly, guidance was given from an academic GP working alongside colleagues from the award-winning charity *healthtalk.org* [37] hosted by the University of Oxford. Finally, expertise was sought from within the MRC START project team which includes clinicians, psychologists and experts in trials.

**Figure 2:**
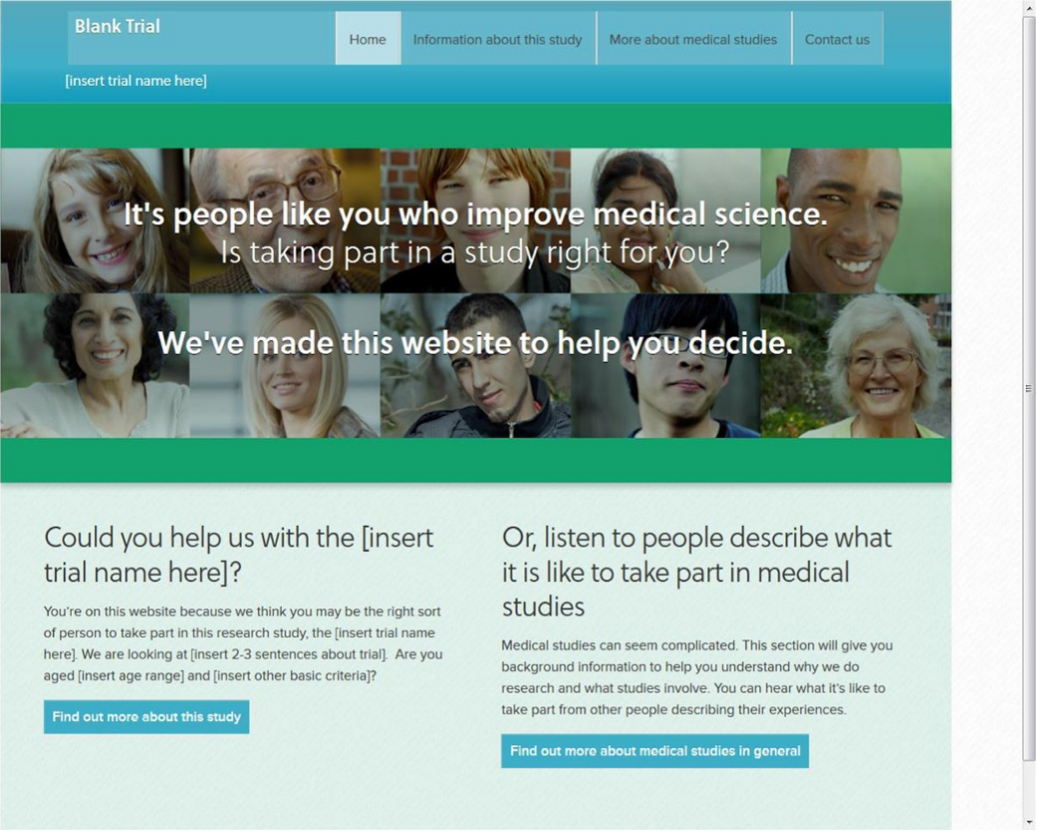
**Web-based platform blank template homepage** [42] **.**

The content was based on relevant theoretical and empirical work about patient decision-making both generally [38, 39] and in trials [40, 41]. It was agreed that the content would not repeat the standard PIS, but be designed specifically to help potential participants make better informed decisions by providing additional information about medical research and trial participation including, where possible, information on previous participants’ experiences of medical studies.To design the web-based platform, the research team developed a draft specification to outline the three components. The first component was a homepage, which needed to be relevant to each specific host trial, giving a brief pitch about the trial to engage potential participants and offering them the options of more information on medical research in general or, finding more about the specific trial (see Figure 2). The second component, generic pages on medical research, included video clips of previous participants describing their experiences taking part in medical research, and cover issues of relevance to potential participants, such as ‘Why get involved?’, ‘Agreeing to take part’, ‘What happens in a study?’, ‘Leaving a study’, and ‘Protecting privacy’.

A small team consisting of two patient and public representatives and a GP from *healthtalk.org* [37], reviewed all *healthtalk.org* video clips relating to patient experience of participation in research. Video clips were selected and edited to illustrate key points from participants talking about their general experiences of medical research. Each video clip was edited by the Patient and Public Involvement representatives, working directly with a GP from *healthtalk.org* [37], on the basis that the clip reflected a non-coercive patient perspective on participating in medical studies.

**Figure 3:**
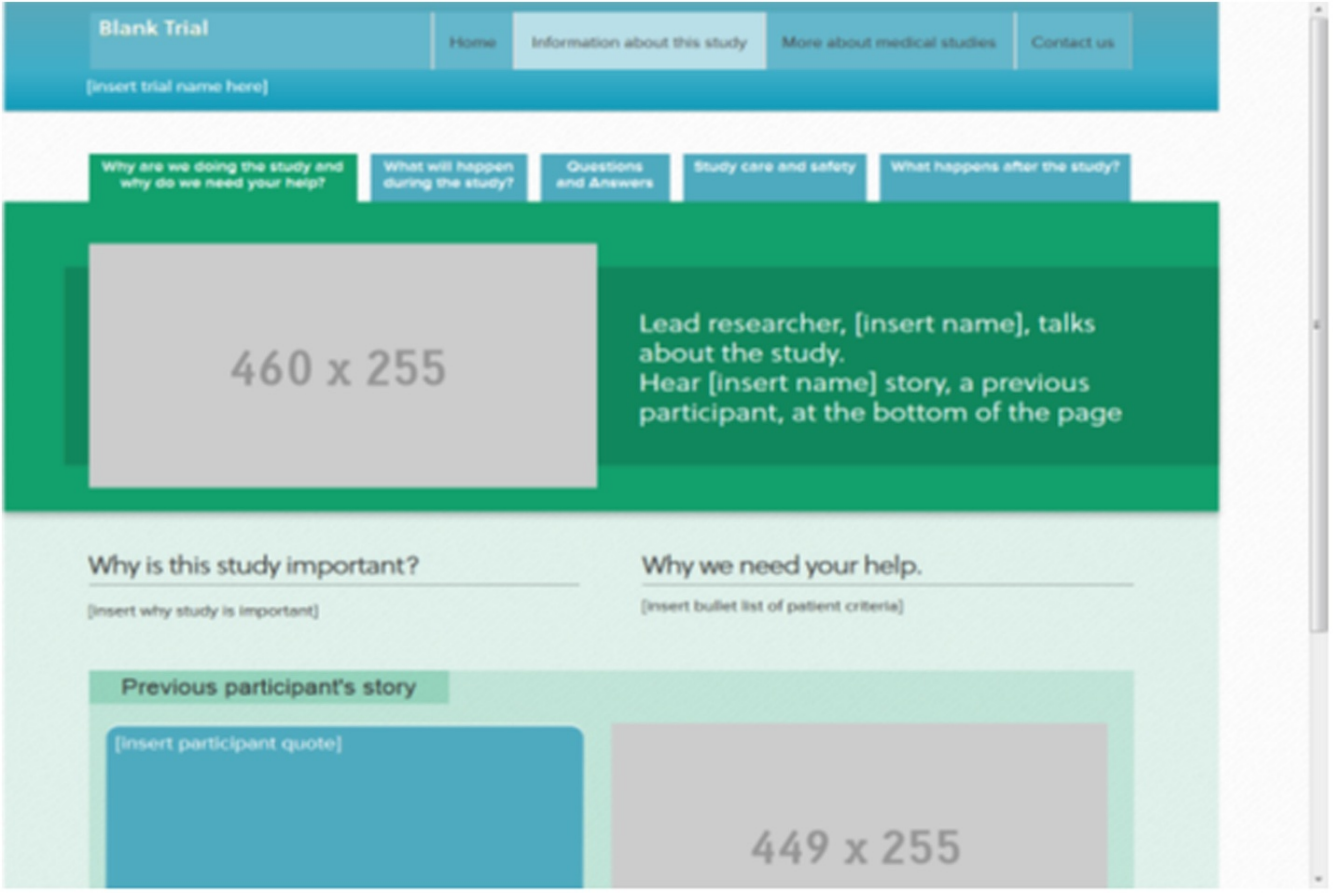
**Web-based platform study-specific pages blank template.**

Specially designed infographics (animated information videos) were developed to accompany the clips in order to visually explain some of the more difficult concepts used in medical research (such as randomization). Each infographic was developed by our digital partners (Reason Digital, Manchester, United Kingdom) based on visual and text material provided by the research team and our Patient and Public Involvement representatives, who all commented extensively on the finished infographic. The third and final component, study-specific pages, were designed as a blank template for host trials to insert their own bespoke content, ideally to outline the purpose of the trial and what it is like to take part. Specifically, the pages have been split to reflect concerns raised by potential participants who wish to make an informed decision on participating in the trial. For example, these pages cover ‘Why are we doing the study and why do we need your help?’, ‘What will happen during the study?’, ‘Questions and answers’, ‘Study care and safety’, and ‘What happens after the study?’The plan is for the START team to work with each host trial team to produce bespoke content for the study-specific section. The bespoke content is intended to convey key points about the study in an accessible form, with templates for video clips of key people involved in the host trial (such as the principal investigator and trial manager) and previous participants of the trial describing their experiences (see Figure 3). The platform, which has been designed to be easily accessed and navigated by potential participants, is web-based and viewable on PCs, laptops, and smartphones. Potential participants will be able to gain access to the multimedia intervention via a URL and a quick response (QR) code specific to the host trial placed at the top of the standard PIS and/or on the patient invitation letter. Potential participants in each host trial will then be randomized to either have access to the multimedia intervention in addition to the standard PIS, or the standard PIS alone.

The MRC START multimedia template can be viewed online [42].

### Work package 3: implementation and analysis

#### Trial recruitment

Our long-term aim is to embed the concept of embedding recruitment studies into the funding and startup process of trials in all healthcare settings, and in publicly and commercially funded trials, in order to ensure that delivery of embedded recruitment studies is a routine part of the delivery of trials in the United Kingdom and elsewhere. In the START program, we will work with major funders, clinical trials units, and the research network infrastructure in the United Kingdom to identify and recruit host trials for embedded studies of the recruitment interventions developed in work package 2.

We plan to recruit six trials for each intervention developed in work package 2 (12 in total). Inclusion criteria for the trial are as follows: trials must be at a stage in their delivery where adoption of the embedded recruitment intervention is feasible (for example, about to apply for research ethics approval, early in the recruitment process, or struggling to recruit); trials must be recruiting from primary care or community settings and involving recruitment procedures amenable to the interventions from work package 2; trials must be approaching at least 800 potential research participants; and there must be agreement in principle to take part in START, to randomize using appropriate procedures, to ensure concealment of allocation, deliver the interventions according to a protocol, and share anonymized data on recruitment with the START team as part of an ongoing collaboration.

The aim will be to recruit a sample of host trials (see ‘Sample Size’ below) that all approach patients using broadly comparable recruitment procedures (such as approaching patients from an existing disease register) but do so in relation to different clinical and health service areas and in different contexts. We do not expect all trials demonstrating an interest to be able to participate in START. We will record the process of negotiation and reasons for participation or non-participation to allow us to understand barriers and facilitators to the recruitment of host trials. We will undertake qualitative interviews with principal investigators and other relevant staff (such as trial managers) of host trials after completion of the embedded trial.

#### Sample size and analysis

Work package 3 is designed to provide two estimates relating to recruitment interventions: (1) the effectiveness of recruitment interventions in the context of a single host trial, and (2) a measure of the variability in effectiveness across multiple host trials.

A large number of trials in primary care and community settings recruit patients with existing health conditions from registers in primary care. For the purposes of the power calculation, we assume that the primary outcome is the proportion of potentially eligible patients who agree to participate following such an invitation. Therefore, the denominator will be the number of patients who are initially invited following screening. It should be noted that this is a larger number than that required for power for an analysis of the primary outcomes within individual trials. For example, a trial which is seeking to recruit 300 patients may need to approach approximately 600 to 1,500, depending on the overall uptake rate. It is the latter, higher figure which is of relevance for the power analysis of START in these contexts.

To provide a conservative sample size estimation, we assume a base response rate of 50% to invitations (although rates in many studies will be significantly lower). We define a significant improvement in recruitment rate as an increase in response of 10%. If individual patients are randomized (for example, to a conventional or optimized participant information sheet), 400 patients per arm would be required to provide 80% power to detect a 10% difference (alpha 0.05). We will plan to restrict START to trials that involve at least this level of recruitment, although we will not exclude studies where interventions can be introduced after recruitment has begun, or where recruitment continues beyond the end of the START study.

It is anticipated that some trials will prefer to use cluster allocation of the START recruitment interventions, to ease the logistical burden on the trial and to ensure the quality of the randomization procedure. We will seek where possible to ensure that the minimum sample size is appropriately inflated in these studies to take into account the cluster allocation, assuming an interclass correlation of 0.02 in line with recent estimates from community studies [43, 44].

To assess effectiveness of recruitment interventions in the context of a single host trial, we will seek to analyze all outcomes of relevance to recruitment, according to the framework developed in work package 1. It is likely that the core analysis will involve dichotomous outcomes (for example randomized or not-randomized) assessed using logistic regression, and controlling for baseline demographic factors. A generic analysis plan is available from our statistician (VM). Where possible, we will work with trials to include additional outcome measures, including patient self-reported outcomes such as satisfaction with information, understanding of the trial, and anxiety. Although recording of data on retention of randomized participants is unlikely to be possible during the timeline of the START study, we will encourage investigators to collect and report data.

In terms of variability across host trials, we will explore this in a meta-analytic framework. The proportions of invited patients recruited into each trial will be entered into a meta-analysis, and the heterogeneity of the intervention effect across trials will be assessed using the I^2^ statistic. If significant heterogeneity is demonstrated, we will explore differences between trials that might explain that variation. The power of any such analyses will be limited because of the small number of trials, but we will explore this issue qualitatively using data collected on the trial, the patient population, and the context of the study. Analyses will be guided by a pre-specified analytic plan. All trial data collected on recruitment and retention facilitated by START will be disseminated in accordance with the MRC START publication strategy during the START funding timeline. Subsequently, data will be captured and reported by the existing Cochrane reviews to ensure their availability outside the START funding and timeline [11, 45].

MRC START has received full ethical approval (Research Ethics Committee: REC Reference: 11/YH/0271) from the National Research Ethics Service (NRES) Committee Yorkshire and the Humber - South Yorkshire, and an MRC START Multimedia Substantial Amendment covering the generic content in the multimedia intervention (REC reference: 11/YH/0271 substantial amendment 2, 31 October 2013). NRES approval will be obtained for each embedded study, via a substantial amendment to the host trial REC.

Patients do not have the opportunity to give informed consent to enter into the embedded recruitment studies. This has been approved by NRES Committee Yorkshire and the Humber - South Yorkshire (REC reference: 11/YH/0271) on the basis that the embedded studies are not withholding information, but are just changing the way it is presented. Each embedded study (standard naming format: ‘MRC START in [insert host trial name]’) will be registered by the host study as a sub-study on the relevant trial registration database.

### Preliminary results

To date, we have completed two rounds of recruitment to START with a third in progress. The recruitment process was designed to test interest in START and explore the feasibility of START becoming self-sustaining. For the first round, trials eligible for START were identified from the Health Technology Assessment (HTA) Programme and Primary Care Research Network (PCRN) databases and sent a START project flyer with a covering letter from either HTA or PCRN encouraging their participation in the START program.

**Figure 4:**
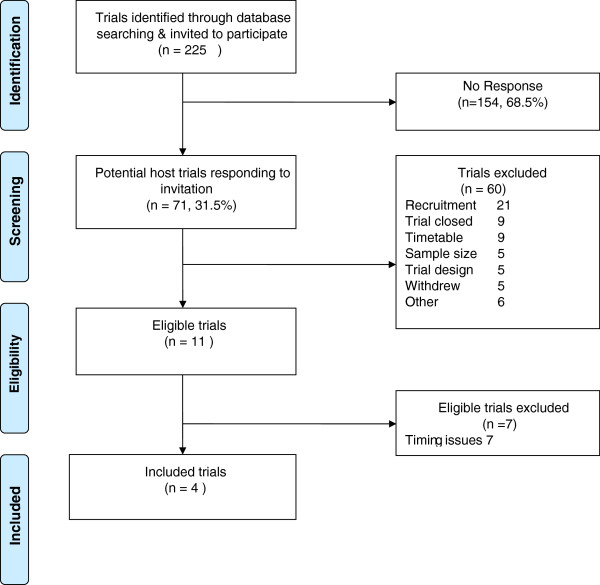
**Round 1 recruitment outcomes.**

For the second round this procedure was repeated and followed up by the START project team who attempted to contact principal investigators up to three times by phone and a further time by follow-up email. For the final rounds of recruitment we are using project flyers, invitation letters, and intensive phone or email follow-up to establish a more comprehensive picture of the barriers or reasons for non-participation.Figure 4 shows the first round of recruitment to the START project. A total of 60 out of 71 potential trials expressing an interest in START were excluded, primarily due to incompatible recruitment methods or aspects of the host trial design, indicating considerable scope for the development of other recruitment interventions. Of the 11 potential trials identified in the first round of recruitment, 4 became hosts to the PIS intervention, and 7 were subsequently lost due to delays in the development of the multimedia intervention. The second and third recruitment rounds are ongoing, with 10 of the 12 trials recruited to date.

There have been some deviations from the protocol in light of challenges encountered during the work. The initial plan was to recruit trials in primary care or community settings and those approaching 800 potential participants. As the project progressed, these criteria were relaxed to include secondary care and smaller trials that were keen to participate and had recruitment procedures amenable to the START interventions. In one trial the existing PIS was of such high quality that, in the opinion of experts within the START team, the PIS development process being evaluated in START would provide few benefits and the trial was excluded from START.

## Discussion

In the short term, the START program is designed to conduct 12 embedded trials of 2 recruitment interventions, in order to provide a robust assessment of the effects of these interventions across a range of trial settings.

The program is also designed to develop capacity for further adoption of the embedded trial methodology, through development of processes (such as those required to gain consent from ethical and other governance committees), resources (such as reporting guidelines and standardized agreements between researchers from host and embedded trials), and knowledge (for example, concerning core barriers to the delivery of embedded trials, and the optimal methods to encourage adoption).

Initial findings show some difficulty engaging with the wider trial community. Of the trials approached in the first round of recruitment, 70% did not respond, although this may reflect the primary care and community focus evident in the START promotional material. Of those trials expressing an interest, the majority were ineligible. Some reasons for the exclusion of potential host trials (such as timetable issues, n = 25 in total) are limitations of the timeline for this research and would not in general be obstacles to testing recruitment interventions by embedded trial methodology. The fact that over a third of potential host trials were excluded due to recruitment method or other trial design issues (n = 26) highlights the need to develop a broader range of recruitment interventions suitable to varying trial recruitment methods. Further adoption and implementation of the START model is likely to be dependent on the ongoing development of tools and interventions that can be tested using the embedded trials approach, including interventions for professionals [11, 46]. However, as noted earlier, this may lead to additional logistical challenges in implementing embedded recruitment studies which may not be fully explored in the current START protocol, with its focus on lower complexity, patient-level interventions. The development of appropriate incentives to encourage adoption of these methods by busy trial teams is also likely to be important. The more intensive follow-up work on barriers to participation, conducted in the second and third recruitment rounds, will inform development in this area.

The START program will in turn contribute to other developing resources around trials methodology, such as Studies Within A Trial (SWAT [47]) and Trial Forge [48], to meet the longer term aim of making the delivery of embedded recruitment trials routine, and to make a substantive contribution to the development of a science of recruitment.

## Trial status

Recruitment began in March, 2012. The second and third recruitment rounds are ongoing, with 10 of the 12 trials recruited to date.

## Abbreviations

HTA: Health technology and assessment
MRC: Medical Research Council
NRES: National Research Ethics Service
PCRN: Primary Care Research Network
PIS(s): Participant information sheet(s)
PRIMER group: Primary Care Research in Manchester Engagement Resource
REC: Research Ethics Committee
START: Systematic Techniques for Assisting Recruitment to Trials.
